# Supplementary material for: P-Glycoprotein (MDR1/ABCB1) Restricts Brain Accumulation of the Novel EGFR Inhibitor EAI045 and Oral Elacridar Coadministration Enhances Its Brain Accumulation and Oral Exposure
Source: Pharmaceuticals (Basel). 2022 Sep 8;15(9):1124. doi: 10.3390/ph15091124 (PMC9505538; doi:10.3390/ph15091124)
Supplement: Supplementary file 1 [file pharmaceuticals-15-01124-s001.zip › pharmaceuticals-1841020-supplementary.pdf]

Supplemental Figures

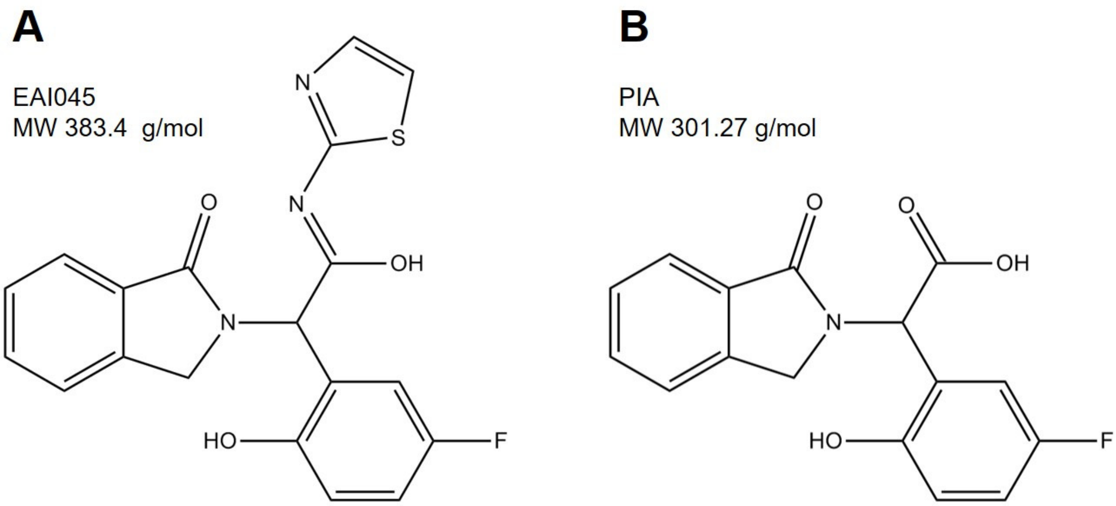

Supplemental Figure S1. Molecular structure of EAI045 (A) and its hydrolyzed metabolite, (5-fluoro-2-hydroxyphenyl) (1-oxo-1,3-dihydro-2H-isoindol-2-yl) acetic acid, abbreviated PIA for (phenyl-(iso)indol-acetic acid) (B). The exact structure of the EAI045 glucuronide is as yet unknown.

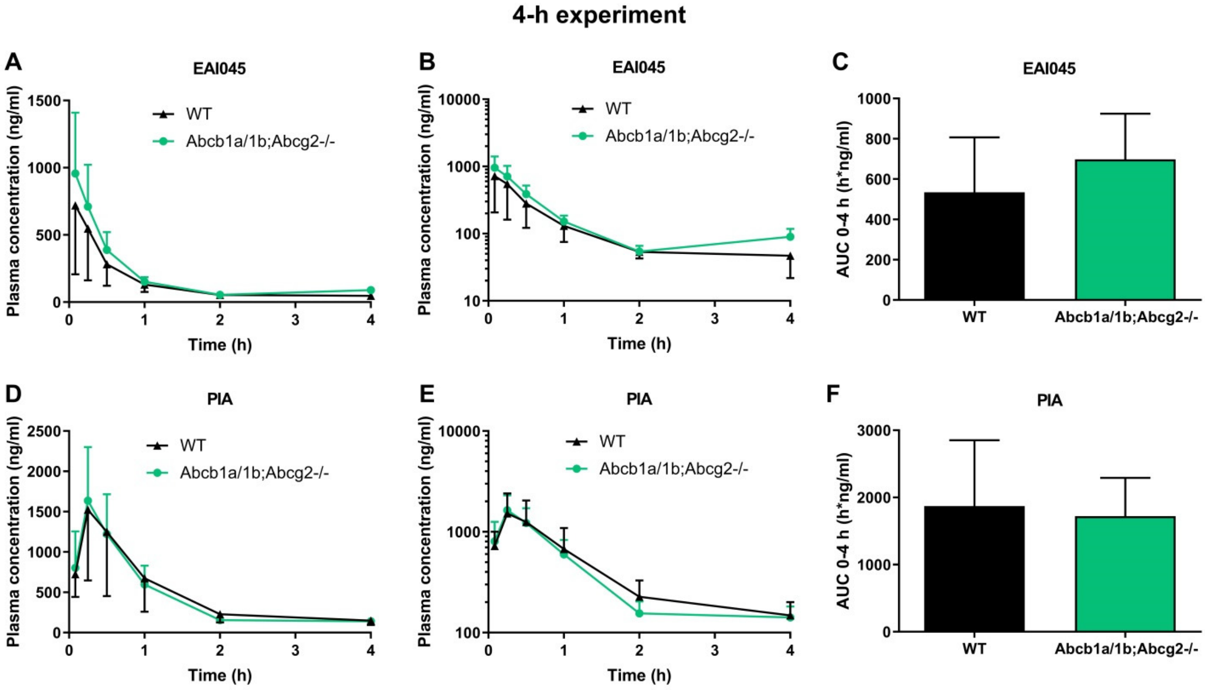

Supplemental Figure S2. Plasma concentration-time curves (A, D), semi-log plots of plasma concentration-time curves (B, E), and $AUC_{0\text{-}4h}$ (C, F) of EAI045 and PIA, respectively, in female WT and *Abcb1a/1b;Abcg2*$^{-/-}$ mice over 4 h after oral administration of 20 mg/kg EAI045. Data are given as mean ± S.D. (n = 6-7).

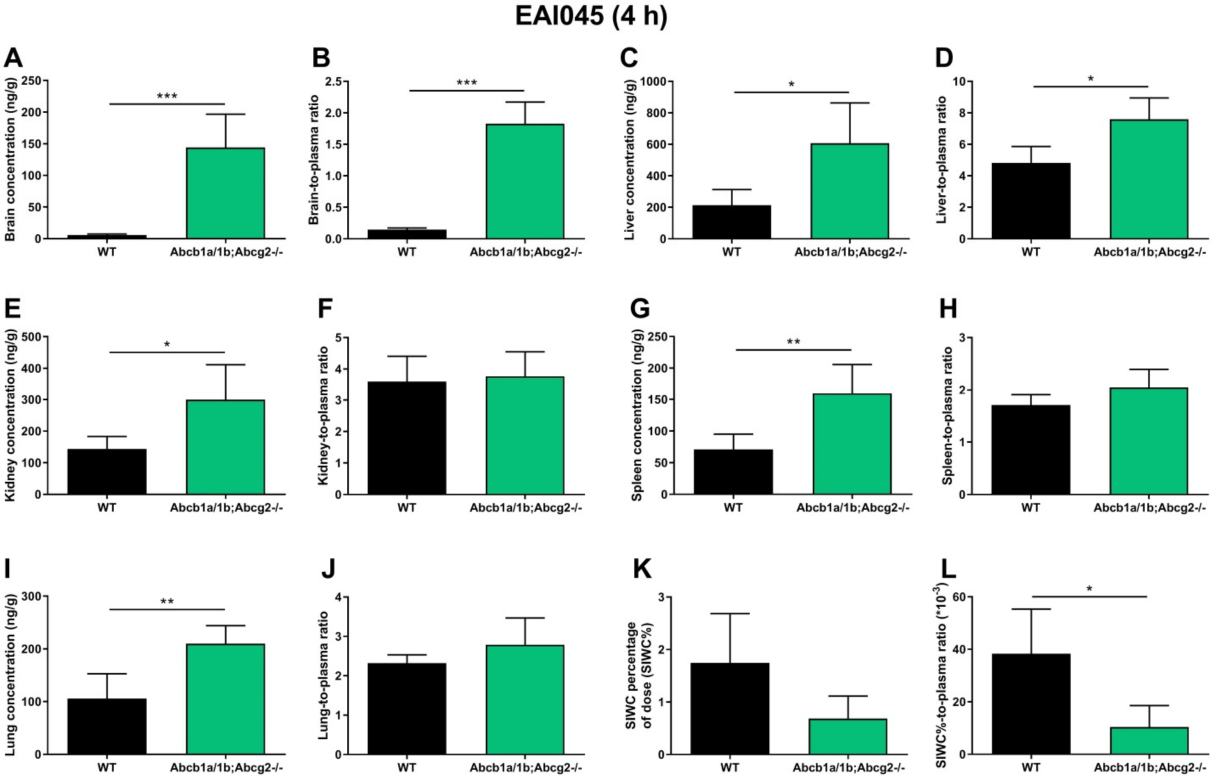

Supplemental Figure S3. Tissue concentration (A, C, E, G, I, K), and tissue-to-plasma ratio (B, D, F, H, J, L) of EAI045 in female WT and *Abcb1a/1b;Abcg2*$^{-/-}$ mice 4 h after oral administration of 20 mg/kg EAI045. Data are given as mean ± S.D. (n = 6-7). *, *P* < 0.05; **, *P* < 0.01; ***, *P* < 0.001 compared to WT mice.

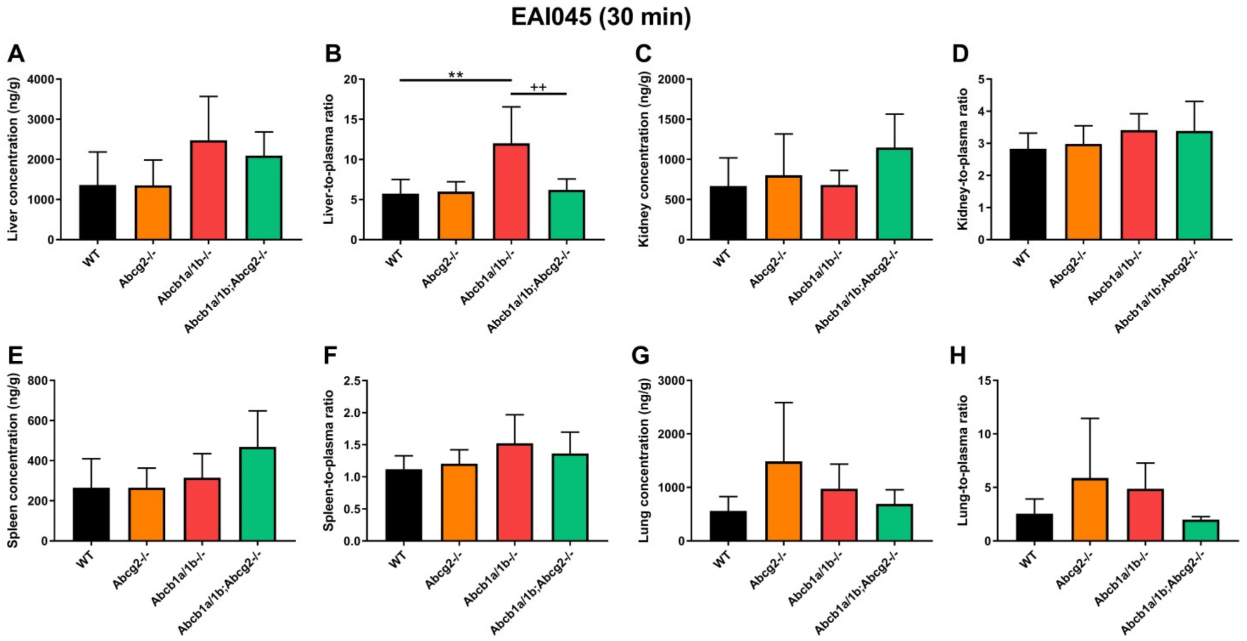

Supplemental Figure S4. Tissue concentration (A, C, E, G), and tissue-to-plasma ratio (B, D, F, H) of EAI045 in WT, *Abcb1a/1b*$^{-/-}$, *Abcg2*$^{-/-}$ and *Abcb1a/1b;Abcg2*$^{-/-}$ mice 30 minutes after oral administration of 20 mg/kg EAI045. Data are given as mean ± S.D. (n = 6-7). *, $P < 0.05$; **, $P < 0.01$; ***, $P < 0.001$ compared to WT mice. $^{+}$, $P < 0.05$; $^{++}$, $P < 0.01$; $^{+++}$, $P < 0.001$ compared to *Abcb1a/1b;Abcg2*$^{-/-}$ mice.

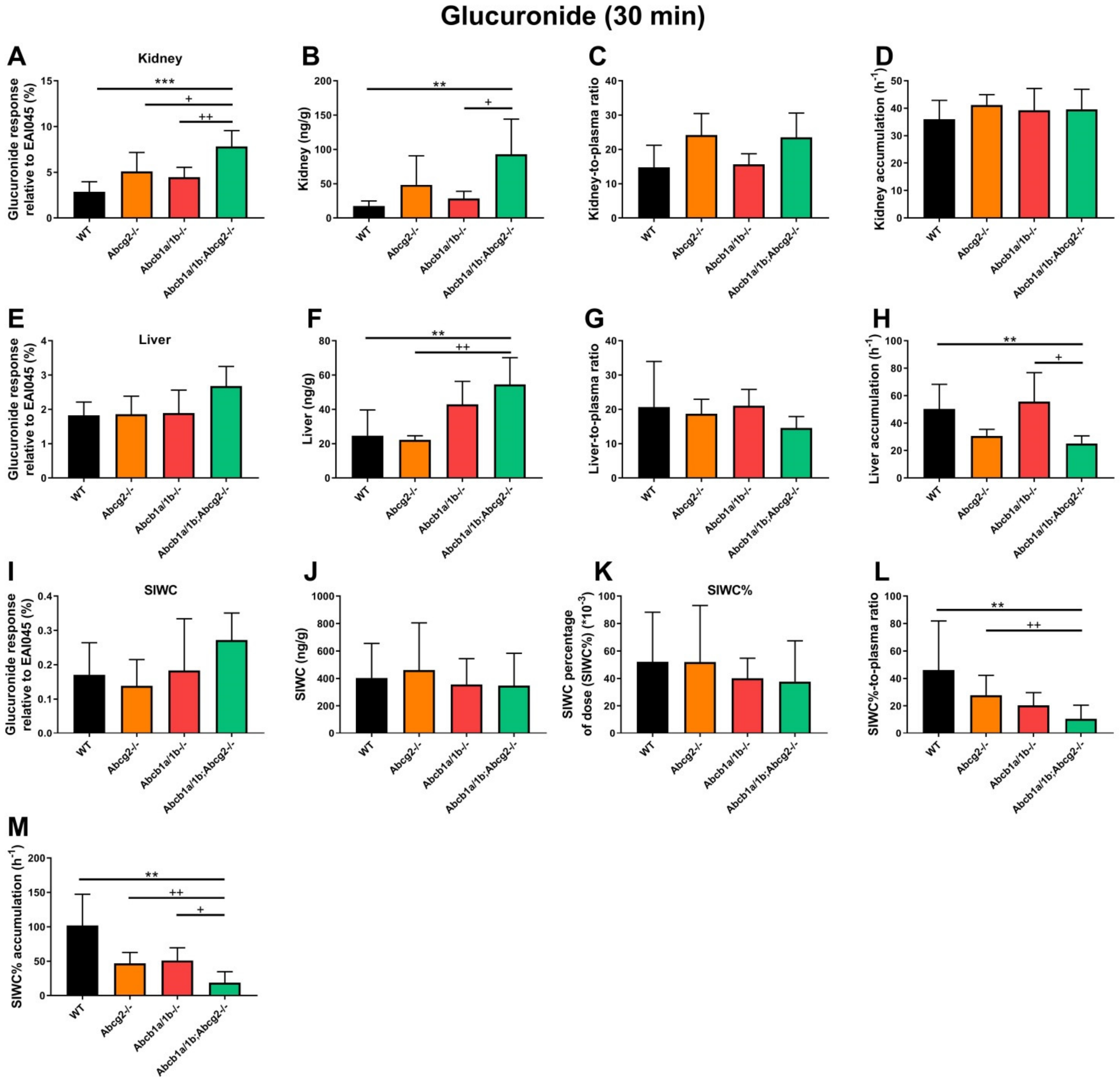

Supplemental Figure S5. EAI045-glucuronide response relative to EAI045 (%) (A, E, I), and accordingly extrapolated glucuronide concentrations (B, F, J, K), glucuronide-to-plasma ratios (C, G, L) and accumulations (D, H, M) in male WT, *Abcb1a/1b*$^{-/-}$, *Abcg2*$^{-/-}$ and *Abcb1a/1b;Abcg2*$^{-/-}$ mice 30 minutes after oral administration of 20 mg/kg EAI045. Data are given as mean ± S.D. (n = 6-7). *, $P < 0.05$; **, $P < 0.01$; ***, $P < 0.001$ compared to WT mice. $^{+}$, $P < 0.05$; $^{++}$, $P < 0.01$; $^{+++}$, $P < 0.001$ compared to *Abcb1a/1b;Abcg2*$^{-/-}$ mice. K-M: data log transformed before applying statistical tests.

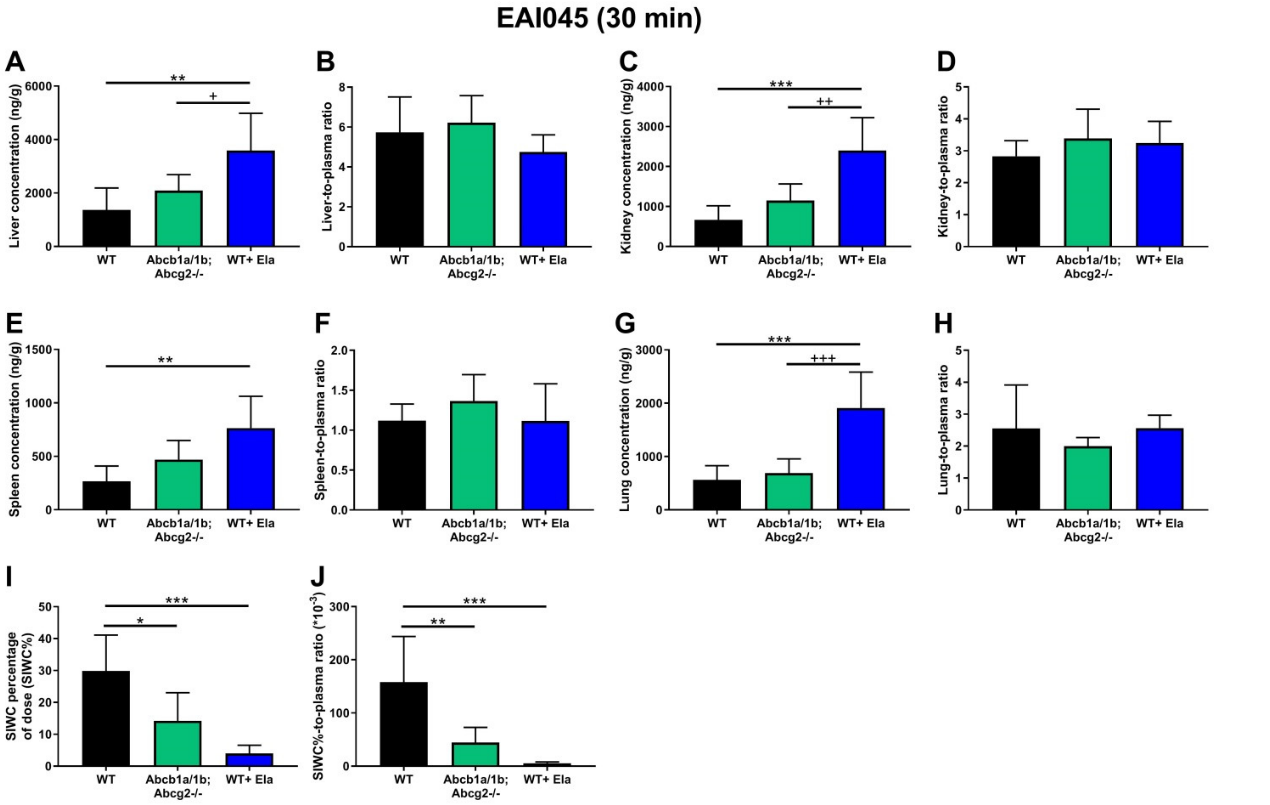

Supplemental Figure S6. Tissue concentration (A, C, E, G) or amount (I), and tissue-to-plasma ratio (B, D, F, H, J) of EAI045 in male WT and *Abcb1a/1b;Abcg2*$^{-/-}$ mice over 30 minutes after oral administration of 20 mg/kg EAI045 without or with co-administration of elacridar (Ela, WT mice only). Data are given as mean ± S.D. (n = 6-7). *, $P < 0.05$; **, $P < 0.01$; ***, $P < 0.001$ compared to WT mice. $^{+}$, $P < 0.05$; $^{++}$, $P < 0.01$; $^{+++}$, $P < 0.001$ compared to *Abcb1a/1b;Abcg2*$^{-/-}$ mice. I-J: data log transformed before applying statistical tests.

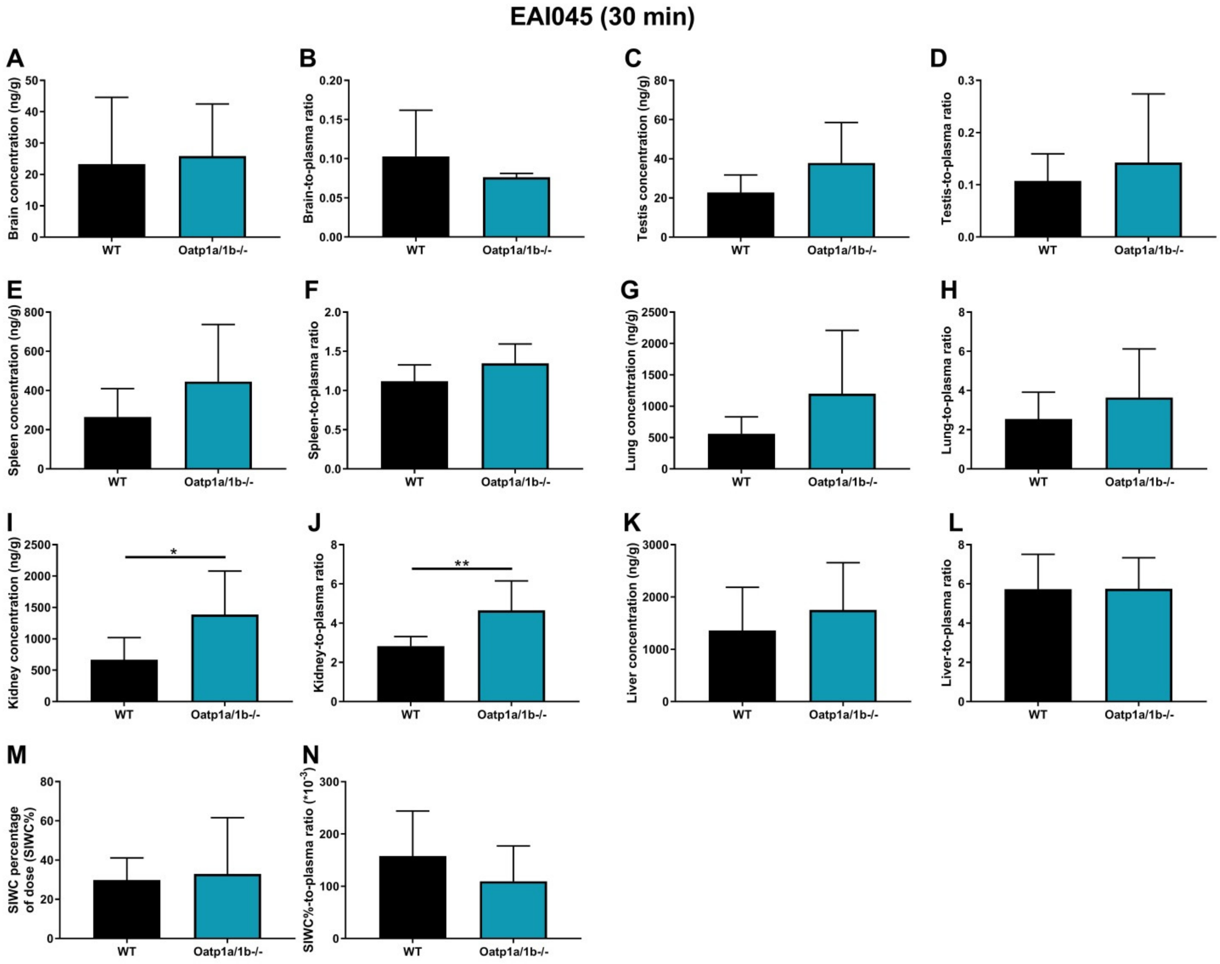

Supplemental Figure S7. Tissue concentrations (A, C, E, G, I, K) or amount (M) and tissue-to-plasma ratios (B, D, F, H, J, L, N) of EAI045 in male WT and *Oatp1a/1b*$^{-/-}$ mice 30 minutes after oral administration of 20 mg/kg EAI045. Data are given as mean ± S.D. (n = 6-7). *, P < 0.05; **, P < 0.01; ***, P < 0.001 compared to WT mice. M-N: data log transformed before applying statistical tests.

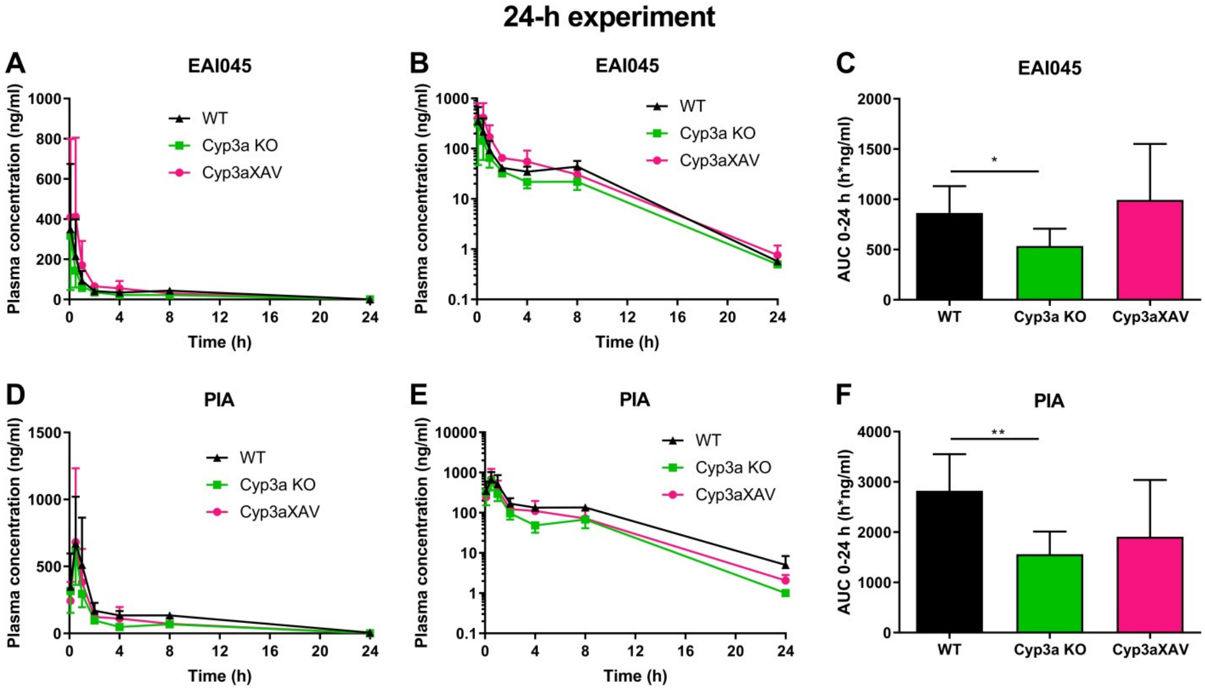

Supplemental Figure S8. Plasma concentration-time curves (A, D), Semi-log plots of plasma concentration-time curves (B, E), and $AUC_{0\text{-}24h}$ (C, F) of EAI045 and PIA in female WT, *Cyp3a*$^{-/-}$, and Cyp3aXAV mice over 24 h after oral administration of 20 mg/kg EAI045. Data are given as mean ± S.D. (n = 6-7). *, P < 0.05; **, P < 0.01; ***, P < 0.001 compared to WT mice.
